# Application of periareolar mastopexy technique for giant phyllodes tumor resection in an adolescent female with breast asymmetry: a case report and literature review

**DOI:** 10.1186/s43046-020-00036-2

**Published:** 2020-06-29

**Authors:** Mahmoud A. Hifny, Mohamed Yousef A., Asmaa Gaber R., Mohamed Abdelshafy Mohamed, Mahmoud Abdelhameid

**Affiliations:** 1 Plastic Surgery Department, Faculty of Medicine, Qena University Hospital, South Valley University, Qena, Egypt; 2 General Surgery Department, Faculty of Medicine, Qena University Hospital, South Valley University, Qena, Egypt

**Keywords:** Giant phyllodes tumor, Breast, Periareolar mastopexy

## Abstract

**Background:**

The phyllodes tumors are rare neoplastic disease which exhibits a benign behavior in adolescents female. After resection of a large benign breast tumor, insufficient breast contour may result with nipple areola complex malposition. As symmetry of the breast is psychologically extremely crucial, especially in adolescents, in such cases, an immediate mammoplasty-like breast reduction or mastopexy technique of the affected breast will be necessary to provide symmetry of the bilateral breasts at the initial surgery.

**Case presentation:**

A 16-year-old woman reported rapid enlargement of a large mass in her left breast over 12 months. The physical examination revealed a huge mass that occupied the lower quadrants of her left breast causing expansion of both the overlying skin and the nipple areolar complex. A biopsy was constant with a benign phyllodes tumor. We have applied a periareolar mastopexy technique to allow tumor resection through exposure incision at the lower half of the outer periareolar circular incision. At the same time, we reduced the expanded skin envelope and mobilized the nipple–areola complex to restore breast symmetry.

**Conclusion:**

The periareolar mastopexy approach provides a wide surgical exposure, allows excision of benign giant breast tumor, and simultaneous restoration of the breast shape with favorable aesthetic results and minimal postoperative scarring.

## Background

The Phyllodes tumor has reported as a rare neoplastic lesion in an female adolescent which account for about 1% of all breast lesions. However, most phyllodes tumor exhibit a benign behavior in children and adolescents [1].

The benign breast disorders such as giant fibroadenomas or phyllodes tumors represent a unique challenge to the reconstructive surgeon, through insufficient breast contour and mal-position of nipple and areola complex that result after resection of a large benign tumor. Therefore, an immediate mammaplasty of the affected breast will be recommended to address breast symmetry [2].

In the following report, we present a case of adolescent female with unilateral giant benign phyllodes breast tumor treated by direct tumor excision through application of periareolar mastopexy technique to correct breast asymmetry in a single stage operation.

## Case presentation

**Fig. 1:**
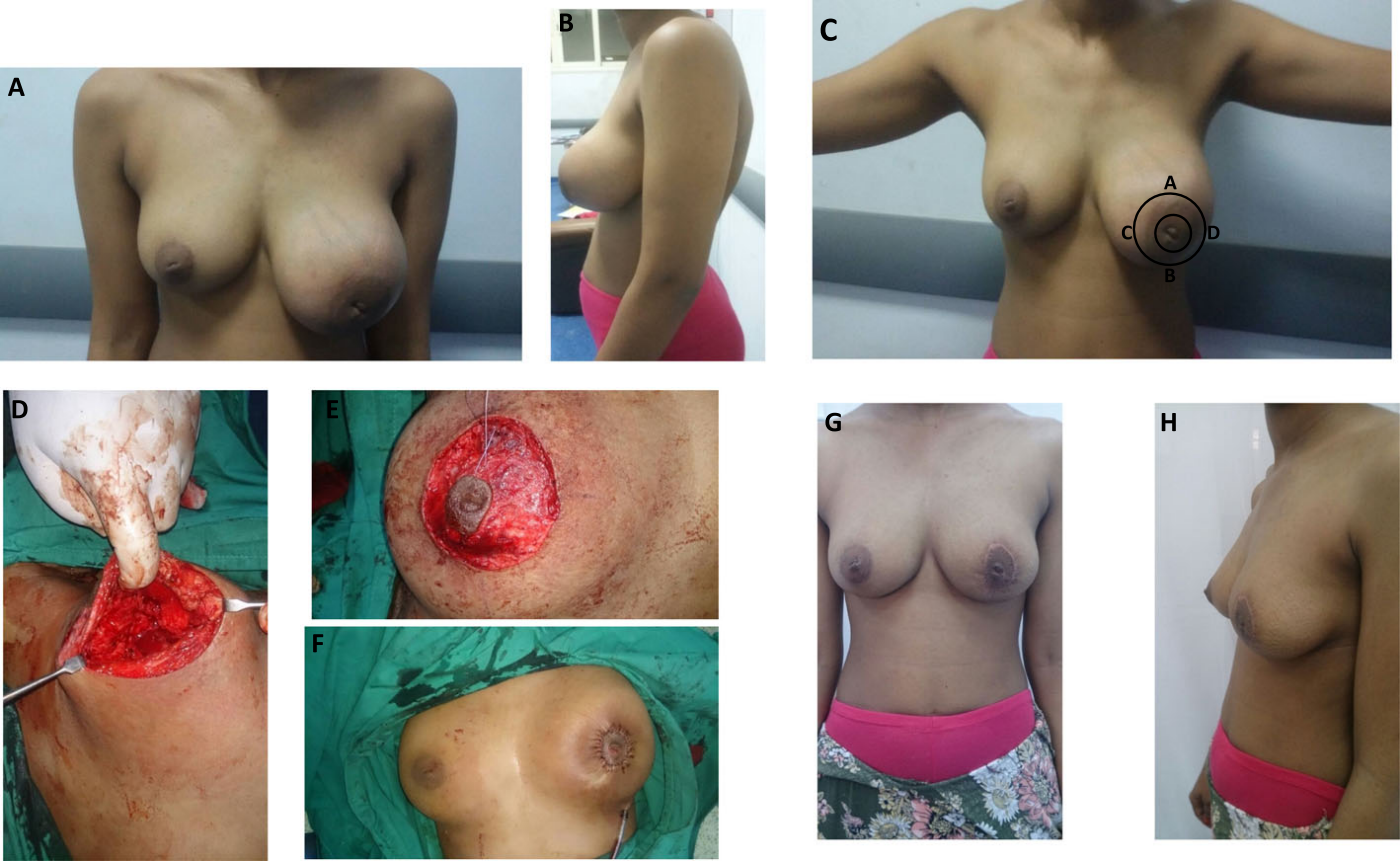
**a** The preoperative appearance of both breasts. The left breast was hugely enlarged by the giant mass. **b** The preoperative lateral view of appearance of the left breast. **c** Preoperative markings for periareolar mastopexy. **(a)** Future superior point of the nipple (nipple to-sternal notch). **(b)** Future inferior point of the nipple (nipple-to-infra-mammary fold distance). **(c)** Medial limit of the nipple. **(d)** Lateral limit of the nipple. All these point was measured in comparison with normal contralateral breast dimensions. **d** After tumor excision through lower half of preplanned outer circular periareolar incision. **e** Round block suture technique to recruit peripheral skin around new areola. **f** Immediate post-operative view. **g, h** Three months postoperative anterior and lateral view of the patient. The breast symmetry and projection have been restored

A 16-year-old woman visited our outpatient clinic and reported rapid enlargement of a large mass in her left breast over 12 months. The physical examination revealed a huge mass that occupied the lower quadrants of her left breast causing expansion of both the overlying skin and the nipple areolar complex (Fig. 1a, b). Ultrasonography revealed lobulated and well-circumscribed solid mass. A fine-needle aspiration biopsy and a Tru-Cut needle biopsy were consistent with a benign phyllodes tumor.

After careful discussion about the best approach to provide an adequate access to the tumor excision as well as addressing the excess skin envelope after tumor excision, the periareolar mastopexy approach was planned. It provides exposure through incision at the lower half of the outer periarolar circular incision for tumor excision. Furthermore, we are able to reduce the expanded skin envelope and mobilize the nipple–areola complex to restore breast symmetry.

### Surgical technique

The technique begins by marking a pair of concentric circumareolar skin incisions. The inner circle diameter represented the areolar diameter and would be reduced in size to be symmetrical with the contralateral breast. The outer circle diameter is determined by standard four key points that are connected to form an outer circular incision pattern. The superior (point A) and inferior points (point B) represent the nipple-to-sternal notch and the nipple-to-infra-mammary fold distance respectively on the breast meridian. The medial and lateral points (point C&D) are determined by the intended skin resection. All these point was measured in comparison with normal contralateral breast dimensions (Fig. 1c). At first, deepithelialization of the entire skin between the inner and outer periareolar incisions was done. Through the preplanned full-thickness incision at the lower half of the outer periareolar circular incision, the phyllodes tumor was easily reached with an adequate and perfect view for resection with sufficient safety margin (Fig. 1d). After a wide local excision of the tumor, no dermoglandular tissue was discarded, and the breast parenchyma was approximated and fixed to the pectoralis fascia. For skin closure, a round block suture technique was placed at the outer skin margin to reduce its diameter to that of the normal areola result in a periareolar scar only (Fig. 1e, f).

The macroscopic appearance of the removed tumor was a well-encapsulated lobulated fibrous mass, measured 12 cm in its maximum dimension. The tumor was histopathologically diagnosed as benign phyllodes tumor consisted of benign epithelial and stromal cells with no obvious mitosis or cellular atypia.

Three months postoperatively, the symmetry of the bilateral breast has been nearly identical in terms of the nipple– areola complex’s position, skin quality, and breast shape (Fig. 1g, h).

## Discussion

The phyllodes tumors are proliferating breast stromal disease which is mostly benign in nature. While the median age of diagnosis is 45 years, however, they are rarely observed in adolescent female, with only about 20 cases reported in the literatures [3].

The mainstay management of benign phyllodes tumor is a wide local surgical excision of the tumor to reduce the local recurrence. Although patients with small- to moderate-sized tumors have had tolerable outcomes with the conservative approach which allowed the stretched skin envelope to retract to its normal form following tumor excision [4]; however, those with larger lesions are left with a displeasing loose ptotic breast [5]^.^ Therefore, several reports [2, 6] have advocated various oncoplastic surgical approach as an effective option to restore breast shape following excision of benign breast tumors.

Yamamoto and Sugihara described a 13-year-old female patient with breast phyllodes treated by inferior pedicle reduction mammaplasty [6]^.^ Yilmaz et al. in 2003 advocated a removal of phyllodes tumor and restoring breast symmetry through a superior pedicle reduction mammaplasty [7]^.^ Moreover, Brier et al. managed a 17-year-old female patient with a phyllodes tumor through single stage excision with reduction mammoplasty [8]. Also, Erginel et al. carried out breast conservative surgery in a 13-year-old female patient with a phyllodes tumor by reposting the areola based on skin flaps and inserting an implant [3].

The mastopexy technique is an operative procedure with different skin excision patterns which correct breast ptosis through elevation of the nipple–areola complex and tightening the skin brassiere [9]. It provides the shortest possible scar pattern as the scar is placed at the border of the pigmented areolar skin and unpigmented breast skin with significant scar camouflage.

With appropriate preoperative planning in such an adolescent female, the application of periareolar mastopexy technique is very useful with excision of the giant phyllodes breast tumor combined with restoration of her breast asymmetry. The tumor is easily resected with an adequate view through a full-thickness incision at the lower half of the preplanned outer periareolar circle. After complete tumor excision, the residual breast tissue is sufficient; therefore, the remaining parenchyma was approximated and fixed to the pectoralis fascia. During closure, the skin at the outer circle was recruited around new areola using round block sutures technique which result in a periareolar scar only. This approach reduces psychosocial morbidity and the need to wait for skin retraction associated with the conservative approach.

## Conclusion

The periareolar mastopexy approach provides a wide surgical exposure, allows excision of benign giant breast tumor, and simultaneous restoration of the breast shape with favorable aesthetic results and minimal postoperative scarring in single stage operation.

## Data Availability

Data sharing is not applicable to this article as no datasets were generated or analyzed during the current study.
